# Do patients with Degenerative Cervical Myelopathy recover as quickly and as well after surgery as patients suffering from other degenerative disorders of the cervical spine?

**DOI:** 10.1016/j.bas.2026.106155

**Published:** 2026-06-30

**Authors:** Francine Mariaux, Anne F. Mannion, Fabio Galbusera, Andrea Cina, François Porchet, Daniel Haschtmann, Markus Loibl, Frank S. Kleinstück, Tamás F. Fekete

**Affiliations:** a Department of Teaching, Research and Development, Spine Centre Division, Schulthess Klinik, Lengghalde 2, Zurich, 8008, Switzerland; b Eidgenössiche Technische Hochschule (ETH), Department of Health Sciences and Technology (D-HEST), ETH Zurich, Lengghalde 2, Zurich, 8008, Switzerland; c Spine Centre, Schulthess Klinik, Lengghalde 2, Zurich, 8008, Switzerland

**Keywords:** Degenerative cervical myelopathy, Patient-rated outcome measures, Spine surgery, Degenerative disorders of the cervical spine, Propensity-score matching, Evolution over time

## Abstract

**Introduction:**

Spinal cord damage in Degenerative Cervical Myelopathy (DCM) makes it a distinct subgroup of degenerative cervical spine disorders, as the central nervous system is affected.

**Research question:**

Are the pattern and extent of change in patient-reported outcomes over time comparable between patients with DCM and those with other degenerative cervical spine disorders?

**Material and methods:**

Pre-operatively and at 3, 12, 24 and 60 months’ follow-up (*Time* factor), patients undergoing cervical spine surgery for degenerative disorders completed a questionnaire comprising the Core Outcome Measures Index (COMI, 0–10), Patient-Acceptable Symptom State (PASS, dichotomised as yes/no), and Global Treatment Outcome (GTO, dichotomised as good/poor). Patients with DCM and controls (*Group* factor) were propensity-score matched based on baseline and surgical variables. Data were analysed using General Estimating Equations.

**Results:**

708 patients (354 DCM, 354 controls; 61.7 ± 10.7 years; 43% female) could be matched and analysed. *Time* was statistically significant for all outcomes (p < 0.01), but *Group* only for GTO (p = 0.001). The *Time × Group* interaction was not significant (COMI p = 0.101; PASS p = 0.062; GTO p = 0.152), indicating similar changes over time for both groups. PASS improved significantly from baseline up to 12 months and COMI up to 24 months. Overall, 7.5% fewer DCM patients than controls reported a good GTO (p = 0.002).

**Discussion and conclusion:**

Recovery patterns over time were similar in DCM and control patients. Differences were observed only for GTO, with fewer DCM patients rating their surgical outcome as good, although COMI and PASS were comparable between the groups.

## Highlights

• Propensity-score matching of surgical patients with degenerative cervical spine disorders: with myelopathy vs. without.
• Patient-rated outcomes were similar between the groups.
• The evolution over time of these outcomes was similar, too.
• But fewer patients with myelopathy rated the outcome of their surgery as good.

## 1 Introduction

Degenerative Cervical Myelopathy (DCM) is a degenerative condition of the cervical spine causing compression of the spinal cord and subsequent neurological dysfunction, resulting in pain, disability and diminished quality of life (Fehlings et al., 2017). Surgery is considered the most efficient treatment to halt disease progression and, in many cases, alleviate symptoms (Fehlings et al., 2017; Mjåset et al., 2022). Many recent studies have concluded that outcomes such as function, pain, disability and quality of life improve significantly after surgery. The timing of recovery after surgery is, however, still unclear. Outcomes have been reported to improve rapidly in the first months after surgery and then reach a plateau (Evaniew et al., 2023; Furlan et al., 2011; Moussellard et al., 2014; El-Hajj et al., 2025). Other studies have reported functional improvement up to several years postoperative (Dijkman et al., 2022; Al-Tamimi, 2013), followed by worsening (Dijkman et al., 2022), suggesting that longer follow-up is required to document the full course of recovery.

Outcomes of surgery can be appraised from different points of view but the importance of providing value to the individual patient is commonly emphasized (Zuckerman et al., 2020). It is now widely accepted that the patient's perspective is essential in evaluating the results of interventions and making medical decisions. As such, outcomes of surgery should not only include cost-effectiveness, mortality rates, functional measures or technical aspects, and thus be assessed by medical staff or insurance companies, but should additionally include the patient's own perception. This can be done using patient-reported outcome measures (PROMs) (Zuckerman et al., 2020; Krauss et al., 2022; Spurgas et al., 2019).

The damage to the spinal cord in DCM makes it a special subgroup within the group of degenerative disorders of the cervical spine, as the central nervous system is affected (Zuckerman et al., 2020; McCartney et al., 2018). For this reason, it is unclear whether the post-operative outcomes of DCM patients, and their evolution over time, are comparable to those of patients with other degenerative cervical disorders not involving the central nervous system. Some studies have investigated the outcome of treatment for different types of degenerative cervical spine disorders but have focused only on specific surgical techniques (Buttermann, 2018; Gornet et al., 2018; Stull et al., 2020; Toci et al., 2023), preventing generalization to all surgical procedures. A very recent study by El-Hajj and colleagues investigated several PROMs of patients with DCM or radiculopathy, pre- and post-operatively (El-Hajj et al., 2025), but the groups showed significant differences in several baseline characteristics, making the post-operative differences between the groups difficult to interpret.

The aim of this study was to investigate the self-reported outcomes of surgery of patients suffering from DCM with those of a matched group of patients suffering from other degenerative disorders of the cervical spine, using PROMs. The goal was to compare between the groups the pattern and extent of change in PROMs over time. We hypothesized that DCM patients would have poorer outcomes as self-reported on PROMs, and that they would experience a slower recovery.

## 2 Materials and methods

### 2.1 Patient selection

An in-house registry, using the framework of the Eurospine Spine Tango Surgery Registry, comprising routinely collected clinical and patient-reported outcome data was searched for patients who had undergone surgery for degenerative disorders of the cervical spine between 2004 and 2019 and was analysed retrospectively.

Exclusion criteria were: neurological comorbidity (other symptomatic disease of the central nervous system), skeletal dysplasia or tumour, aged under 18 years, both lumbar and cervical surgery performed at the same time, fusion-only surgery (no decompression performed), surgery at any level above C2, disc prostheses, current surgery being a revision procedure, inability to understand the languages in which the questionnaire (see later) was available.

All patients with a diagnosis of DCM were included in the myelopathy cohort. A comparative control group from the remaining identified patients was created using propensity-score matching (more details below), to reduce potential selection bias in subsequent analysis. Classification of the main pathologies of the control group was made using the “Cervical Diagnosis groups in degenerative disease” algorithm of Spine Tango (EUROSPINE, 2025).

### 2.2 Data collection

The clinical data were retrieved from the clinic's information system and from the institutional registry. The patient questionnaire used was the Core Outcome Measures Index (COMI) (Mannion et al., 2009). This is a multidimensional 7-item instrument covering the key outcome domains in patients with spinal disorders: pain intensity (axial and peripheral pain, measured separately); function; symptom-specific well-being; general quality of life; and social and work disability.

Patients were asked to fill out the COMI pre-operatively (baseline) and at 3-months’ (3mo), as well as 12-months' (12mo), 24-months' (24mo) and 60-months’ (60mo) follow-up (FU). At all follow-up time points, in addition to the COMI, an item assessing global treatment outcome (GTO) was completed (see below).

### 2.3 Outcomes

Three patient-reported outcome scores were used in the analysis:

• The **COMI** score, calculated from the 7 sub-items described above, ranging from 0 (best) to 10 (worst state) (Mannion et al., 2025a).
• The Patient-Acceptable Symptom State (**PASS**), given by the symptom-specific well-being item of the COMI. This item enquires: “If you had to spend the rest of your life with the symptoms you have right now, how would you feel about it?” Possible answers are on a 5-point Likert scale, from “very satisfied” (1) to “very dissatisfied” (5). Patients responding “very satisfied” or “somewhat satisfied” (i.e., answers 1 or 2) are considered to be in an acceptable symptom state (being in PASS) (Mannion et al., 2025a).
• The Global Treatment Outcome (**GTO**), which enquires “Overall, how much did the surgery help your neck problem?” with a 5-point response scale from “helped a lot” (1) to “made things worse” (5). The answers were dichotomised into “good-GTO " (answers 1 and 2) and “poor-GTO” (answers 3, 4 and 5) (Mannion et al., 2025a).

### 2.4 Propensity score (PS) matching

Direct comparisons of DCM patients and patients suffering from other degenerative pathologies of the spine are difficult because their baseline characteristics often differ, and DCM patients tend to have more invasive surgeries (Pierce et al., 2019). To counter this and balance potential confounders, patients were matched using propensity scores. Variables used to match patients were: age, sex, ASA grade, COMI baseline score, modified Mirza invasiveness score of the procedure (Holzer et al., 2021), any previous surgery at same or adjacent level (yes/no).

Propensity score matching was performed with a custom Python script based on the PsmPy library to balance patient characteristics between the two groups using logistic regression with a caliper width of 0.2 standard deviations.

### 2.5 Statistical analysis

Mann-Whitney U tests, Chi-square tests and Fisher's exact tests were used to compare the patient groups' characteristics before and after PS-matching.

For the main analysis of outcomes, Generalized Estimating Equations (GEEs) were used. GEEs are used for longitudinal data and are similar to mixed-effects models. The only difference is that GEEs model average responses across the population rather than estimating individual responses. They can model both continuous (COMI score) and binary (PASS and GTO) outcomes by specifying a different link function in the model formula, while properly accounting for the within-subject correlations inherent in longitudinal data. The link functions consisted of a Gaussian link for the COMI score and a logistic link for binary outcomes. The formula for the model can be expressed as:

Outcome=Group+Time+Group∗Time+ID

where *Group* is the patient group (DCM or control), *Time* is the time point when the outcome was collected (Pre, 3mo 12mo, 24mo or 60mo), and *Outcome* is the outcome of interest (COMI score, GTO or PASS). The interaction term *Group ∗ Time* was specified to find any difference between the two groups in the change in outcome over time. Additionally, patient ID (*ID)* was specified in the model to account for potential unmeasured correlations among repeated observations from the same individual.

Depending on the significance of *Time, Group,* and their interaction, post-hoc analyses were performed to identify any pairwise differences. If the interaction term was not significant, post-hoc tests were run on all patients (both groups pooled) to investigate the T*ime* factor and on the data from all time points pooled to investigate the differences between the groups. For the continuous outcome, 95% confidence intervals (CIs) were estimated by using the standard errors of the model. For binary outcomes, CIs for estimated proportions were obtained by using bootstrapping to account for the non-linear logistic transformation.

The Mann-Whitney, Chi-square and Fisher's exact tests were run in GraphPad Prism version 9.5.1 for Windows (GraphPad Software, San Diego, California USA). The GEE model was carried out using a Python script based on the statsmodels library.

## 3 Results

### 3.1 Descriptive statistics

**Fig. 1:**
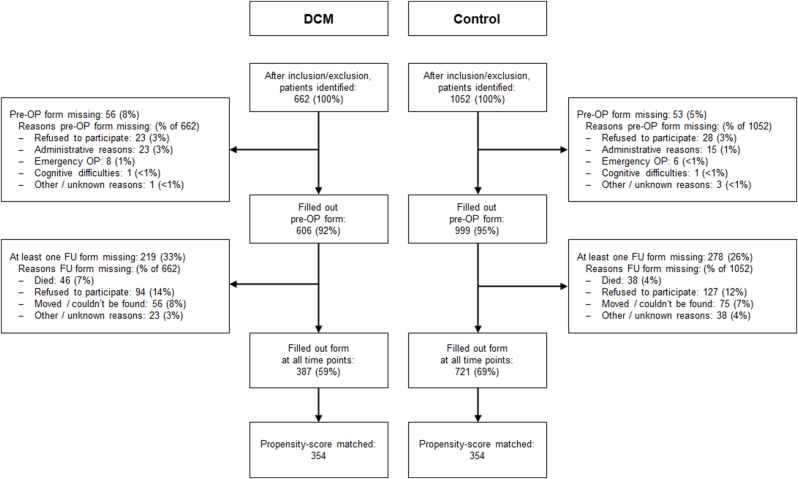
Flow chart of patient inclusion DCM: Degenerative Cervical Myelopathy; FU: Follow-up.

The formation of the final study groups is summarized in Fig. 1.

Of 662 patients who underwent surgery for DCM over the time period in question, 606 (91.5%) filled out the COMI pre-operatively. The FU forms were filled out by 593 (89.6%) at 3 months, 573 (86.6%) at 1 year, 537 (81.1%) at 2 years, and 455 (68.7%) at 5 years. A total of 387 (58.5%) patients filled out the form at all time points and were used for the propensity-score matching.

Over the same time period, of 1052 patients with other degenerative disorders of the cervical spine who underwent surgery, 999 (94.9%) filled out the COMI pre-operatively. The FU forms were filled out by 983 (93.4%) at 3 months, 946 (89.8%) at 1 year, 910 (86.4%) at 2 years, and 830 (78.8%) at 5 years. A total of 721 (68.5%) patients had a complete set of forms from all time points and were used for propensity-score matching. The main reasons for not completing a COMI were the same in both groups and are detailed in Fig. 1.

**Fig. 2:**
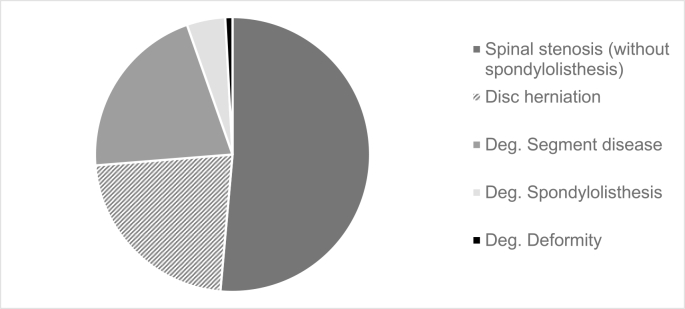
Main pathologies of the control group Deg.: degenerative.

From the 387 myelopathy patients and the 721 patients in the control group with a full set of questionnaires, 354 patients in each group were matched by propensity score, leading to 708 patients (61.7 ± 10.7 years, 43% female) being included in the analysis. Characteristics of the two patient cohorts, both before and after matching, can be seen in Table 1. The patients in the control group were mainly suffering from spinal stenosis (51.4%), disc herniation (22.3%) or degenerative segment disease (20.9%) (Fig. 2). The matched cohorts did not differ in baseline and operative characteristics other than for surgical access, with DCM patients having a significantly higher rate of interventions with posterior and combined approaches than did the control cohort (Table 1).

**Table 1:** Baseline and surgical characteristics of the patient groups, before and after matching.

		Before matching			After matching		
		DCM	Control	p-value	DCM	Control	p-value
N patients		387	721		354	354	

Age (years) (mean (SD))		62.3 (11.2)	57.9 (10.9)	**<0.001**	61.6 (11.2)	61.8 (10.1)	0.903
Sex: female (N (%))		162 (41.9%)	362 (50.2%)	**0.008**	152 (42.9%)	152 (42.9%)	>0.999
ASA grade	mean (SD)	2.3 (0.6)	2.1 (0.6)	**<0.001**	2.3 (0.5)	2.3 (0.5)	0.537
	categories (N (%))			**<0.001**			0.474
	1	27 (7.0%)	71 (9.8%)		27 (7.6%)	19 (5.4%)	
	2	223 (57.8%)	490 (68.0%)		211 (59.6%)	217 (61.3%)	
	3	133 (34.4%)	155 (21.5%)		114 (32.2%)	113 (31.9%)	
	4	3 (0.8%)	5 (0.7%)		2 (0.6%)	5 (1.4%)	
*Missing: N (% of the group)*		*1 (0.3)*	*0 (0.0)*		*0 (0.0)*	*0 (0.0)*	
BMI	mean (SD)	25.9 (4.3)	25.7 (4.7)	0.154	25.7 (4.3)	26.1 (4.7)	0.461
*Missing: N (% of the group)*		*54 (14.0)*	*85 (11.8)*		*54 (15.2)*	*44 (12.4)*	
BMI category	N (%)			**0.019**			0.275
	<20 kg/m2	13 (3.8%)	38 (5.8%)		13 (4.2%)	19 (6.0%)	
	20-25 kg/m2	148 (42.9%)	324 (49.7%)		137 (43.9%)	142 (44.5%)	
	26-30 kg/m2	140 (40.6%)	201 (30.8%)		124 (39.7%)	106 (33.2%)	
	31-35 kg/m2	37 (10.7%)	67 (10.3%)		32 (10.3%)	41 (12.9%)	
	>35 kg/m2	7 (2.0%)	22 (3.4%)		6 (1.9%)	11 (3.4%)	
*Missing: N (% of the group)*		*42 (10.9)*	*69 (9.6)*		*42 (11.9)*	*35 (9.9)*	
Smoker: yes (N (%))		95 (29.1%)	196 (32.0%)	0.374	87 (29.6%)	76 (25.7%)	0.312
*Missing: N (% of the group)*		*60 (15.5)*	*109 (15.1)*		*60 (16.9)*	*58 (16.4)*	
Any previous surgery at same or adjacent segment: yes (N (%))		25 (6.5%)	60 (8.3%)	0.289	23 (6.5%)	24 (6.8%)	>0.999
COMI baseline score (mean (SD))		5.9 (2.5)	7.0 (2.1)	**<0.001**	6.2 (2.4)	6.2 (2.2)	0.967

Surgical access	N (%)			**<0.001**			**0.018**
	Anterior	308 (79.6%)	639 (88.6%)		286 (80.8%)	313 (88.4%)	
	Posterior	46 (11.9%)	60 (8.3%)		42 (11.9%)	27 (7.6%)	
	Combined	33 (8.5%)	22 (3.1%)		26 (7.3%)	14 (4.0%)	
Surgery type	N (%)			0.693			0.265
	Decompression only	25 (6.5%)	42 (5.8%)		25 (7.1%)	17 (4.8%)	
	Decompression and any fusion or stabilization	362 (93.5%)	679 (94.2%)		329 (92.9%)	337 (95.2%)	
Modified Mirza score	mean (SD)	8.4 (4.4)	7.1 (3.5)	**<0.001**	8.0 (3.9)	7.9 (4.0)	0.545
Complication during surgery or hospital stay (N (%))		41 (10.8%)	52 (7.2%)	0.052	37 (10.7%)	30 (8.5%)	0.369
*Missing: N (% of the group)*		*7 (1.8)*	*0 (0.0)*	*0 (0.0)*	*7 (2.0)*	*0 (0.0)*	

Note: Univariate tests across study groups include Mann-Whitney tests for continuous variables and chi-square tests or Fisher's exact test for categorical variables.

Bold p values indicate statistical significance (p < 0.05).

DCM: Degenerative Cervical Myelopathy; ASA: American Society of Anesthesiologists Physical Status; BMI: Body Mass Index; COMI: Core Outcome Measures Index.

### 3.2 COMI

**Fig. 3:**
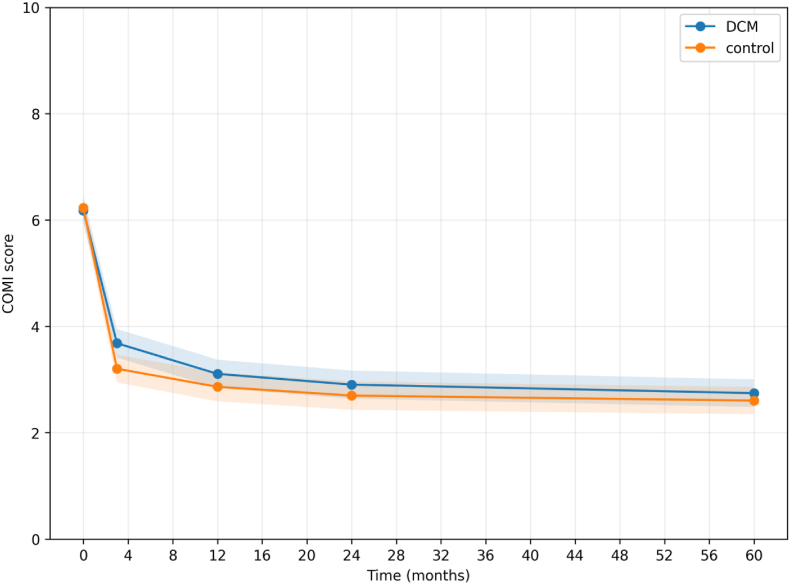
COMI score for each group (mean with 95% CI) DCM: Degenerative Cervical Myelopathy; COMI: Core Outcome Measures Index.

There was no significant difference between the groups for the COMI score over all time points considered together (*Group* factor, p = 0.171) or for the pattern of change over time (interaction, p = 0.101) (Table 2). For both groups together, COMI scores changed significantly over time (*Time* factor, p < 0.001), improving a lot (−2.8 (95% confidence interval (CI), −3.0 to −2.6); p < 0.001)) by 3mo, and showing a less extensive but still significant change from 3mo to 12mo (−0.5 (CI, −0.6 to −0.3); p < 0.001), and from 12mo to 24mo (−0.2 (CI, −0.3 to −0.0); p = 0.0171). There was no statistically significant change from 24mo to 60mo (Table 3 and Fig. 3).

**Table 2:** Overall effects of time of assessment, group, and their interaction on each outcome measure (GEE analyses).

Outcome	Model term	Chi^2^	Degrees of freedom	p-value
**COMI**	**Time**	1362.7	4	**<0.001**
	Group	1.9	1	0.171
	Time x Group	7.7	4	0.101
**PASS**	**Time**	372.8	4	**<0.001**
	Group	0.09	1	0.764
	Time x Group	9	4	0.062
**GTO**	**Time**	11.6	3	**0.009**
	**Group**	10.4	1	**0.001**
	Time x Group	5.3	3	0.152

Bold p values indicate statistical significance (p < 0.05).

COMI: Core Outcome Measures Index; PASS: Patient-Acceptable Symptom State; GTO: Global Treatment Outcome.

**Table 3:** Scores/proportions for and difference between each timepoint for all 3 outcomes.

Timepoint	COMI			In PASS			Good-GTO		
	Score (mean (95% CI))	Difference (mean (95% CI))	p-value∗	Proportion (%] (mean (95% CI))	Difference (mean (95% CI))	p-value∗	Proportion (%] (mean (95% CI))	Difference (mean (95% CI))	p-value∗
Pre	6.2 (6.0 to 6.4)			7.3 (5.7 to 9.4)					
		−2.8 (−3.0 to −2.6)	**<0.001**		44.6 (40.6 to 48.5)	**<0.001**			
3mo	3.4 (3.3 to 3.6)			51.9 (48.3 to 55.7)			81.2 (78.1 to 84.1)		
		−0.5 (−0.6 to −0.3)	**<0.001**		5.6 (1.6 to 9.4)	**0.02**		−2.8 (−5.6 to 0.0)	0.233
12mo	3.0 (2.8 to 3.2)			57.5 (53.9 to 61.1)			78.4 (75.4 to 81.3)		
		−0.2 (−0.3 to 0.0)	**0.017**		2.1 (−1.6 to 5.5)	0.324		3.4 (1.1 to 6.0)	**0.043**
24mo	2.8 (2.6 to 3.0)			59.7 (56.0 to 63.2)			81.8 (78.9 to 84.5)		
		−0.1 (−0.3 to 0.0)	0.082		2.5 (−0.9 to 6.0)	0.324		0.7 (−1.8 to 3.2)	1.000
60mo	2.7 (2.5 to 2.9)			62.2 (58.5 to 65.8)			82.5 (79.6 to 85.1)		

COMI (Core Outcome Measures Index): Scores and score differences between all consecutive timepoints (mean, with 95% confidence interval); PASS (Patient-Acceptable Symptom State): Proportions of patients being in PASS and differences between all consecutive timepoints (mean, with 95% confidence interval); Good-GTO: (Good Global Treatment Outcome): Proportions of patients reporting GTO as good (operation “helped” or “helped a lot”) and differences between all consecutive timepoints (mean, with 95% confidence interval).

A negative value represents a decrease from earlier to later timepoint.

∗p-values from post-hoc analyses; bold indicate statistical significance (p < 0.05).

### 3.3 PASS

**Fig. 4:**
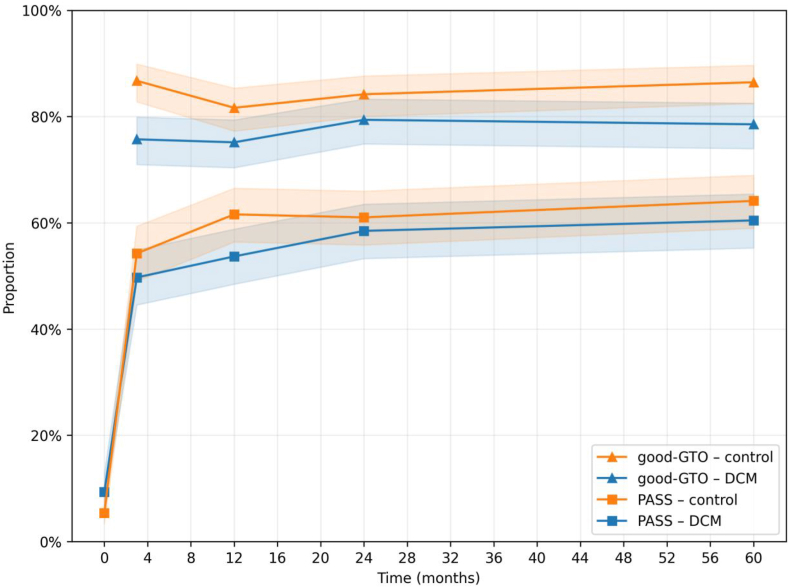
Proportions of patients in PASS or reporting good-GTO (mean and 95% CI) DCM: Degenerative Cervical Myelopathy; GTO: Global Treatment Outcome; PASS: Patient-Acceptable Symptom State.

The proportion of patients in PASS did not differ significantly between the groups (p = 0.764). It increased significantly over time (p < 0.001), in a similar pattern for both groups (interaction non-significant, p = 0.062). The percentage of patients reporting being in PASS was very low pre-operatively (7.3%) and was significantly higher at all FU time points (all p < 0.001). After a large increase of 44.6% (CI, 40.6-48.5%; p < 0.001) from Pre to 3mo, the proportion of patients in PASS increased by a further 5.6% (CI, 1.6-9.4%; p = 0.02) from 3mo to 12mo but not later (Table 2,Table 3 and Fig. 4).

### 3.4 GTO

GTO reflects the patient's perception of the improvement in their condition compared with their preoperative status. As such, it is only enquired about at FU time points. The factors *Group* and *Time* were both statistically significant (p = 0.001 and p = 0.009, respectively), but not the interaction (p = 0.152). Overall, 7.5% fewer patients in the DCM group reported a good-GTO compared with the control group (p = 0.002); depending on the time point in question, 75-79% of the DCM patients versus 81-86% of the control group reported that the operation helped or helped a lot. A closer look at the differences over time for the whole patient group together showed a small but significant increase in the prevalence of good-GTO between 12mo and 24mo (+3.4% (1.1-6.0% CI); p = 0.043) (Table 2,Table 3, Fig. 4).

## 4 Discussion

### 4.1 Summary of main findings

Our analysis on propensity-score-matched patients showed that both myelopathy patients and patients suffering from other degenerative disorders of the cervical spine improved markedly after surgery. None of the PROMs analysed showed any difference in the evolution over time between the groups, refuting our hypothesis that DCM patients would need longer to recover after surgery than control patients. The groups had similar COMI scores and PASS prevalence, but fewer patients in the DCM group estimated that their surgery had helped compared with the control group (lower proportion of good-GTO).

### 4.2 COMI

After surgery, the COMI score of both cohorts decreased significantly up to 24 months, with a mean decrease of 3.4 points for all patients, then remained stable up to 60 months. Two other studies used the COMI to investigate the outcome of surgery for degenerative disorders of the cervical spine over a FU longer than 12mo (Grob et al., 2010; Staub et al., 2016) and they showed a comparable reduction in score (3.7 points). A PROM that has been used more frequently in the literature is the Neck Disability Index (NDI) (Vernon et al., 1991). A crosswalk between the NDI and COMI was recently developed, allowing the scores of one to be interpreted in terms of the other (Mannion et al., 2025b). Using this crosswalk, we calculated that the reductions in NDI scores reported in previous studies equated to reductions in COMI scores of 2.7-4.5 points at 12mo FU (Evaniew et al., 2023; El-Hajj et al., 2025; Krauss et al., 2022; Gornet et al., 2018; Stull et al., 2020; Toci et al., 2023; Johansen et al., 2024) (compared with 3.2 points in the present study) and 2.7-4.9 points at 24mo FU (El-Hajj et al., 2025; Gornet et al., 2018) (compared with 3.4 points in our study). Differences in surgical approaches, patients’ baseline characteristics/symptom severity and study design could all account for the slight differences in these absolute score-changes.

When comparing the absolute COMI scores between the two groups averaged over all time points, no significant differences were found and the groups also showed comparable improvements over time. This is consistent with the finding of two previous studies comparing (other) PROMs in myelopathy and radiculopathy patients (Gornet et al., 2018; Stull et al., 2020), but is partially in disagreement with other studies which reported either comparable outcomes at baseline but not post-operatively (El-Hajj et al., 2025; Stull et al., 2020; Toci et al., 2023), or the other way around (Toci et al., 2023), resulting in different magnitudes of improvement between the patient cohorts. Again, differences in surgical procedures, baseline characteristics and, importantly, the lack of matching of patient cohorts at baseline could explain the differences between the studies.

While our work showed small but significant changes in COMI score at all consecutive time points beyond 3mo up to 24mo, other studies observed a plateau in the evolution of (other) PROMs at 12mo (Evaniew et al., 2023; El-Hajj et al., 2025), and yet another measured a continuous improvement up to the last FU time point at 5y (Al-Tamimi, 2013). However, consistently in all studies, the magnitude of change was greatest between baseline and the first FU time point and then decreased with each further time interval. Differences as to when the score failed to (statistically significantly) improve any further could be the consequence of differences between the studies in their drop-out rates, choice of PROM, statistical tests employed, and size of the cohorts.

### 4.3 PASS

Being in an “acceptable symptom state” (in PASS) indicates that a patient would be satisfied to live the rest of their life with their current symptoms. In contrast to the GTO, which assesses whether the patient feels *better* after treatment, the PASS indicates whether the patient feels *well*. We are unaware of any previous studies investigating this particular concept in cervical spine surgery patients. Although the proportion of patients in PASS increased dramatically from pre-operative to all post-operative time points, as would be expected, it did not reach post-operative values higher than 50-64%. These values may seem low, but they are nonetheless higher than those reported after surgery for lumbar degenerative disorders (44-55% at 12mo) (Fekete et al., 2016; Mannion et al., 2018).

### 4.4 GTO

GTO was consistently rated highly, with 75-79% of all DCM patients and 81-87% of control patients rating their GTO as good (operation helped or helped a lot) over all FU time points. The lower value in the DCM than the control group was statistically significant. Lower rates of patient-rated success and satisfaction have been observed before for DCM patients compared with patients suffering from other degenerative disorders of the cervical spine (Mjåset et al., 2020, 2022; Krauss et al., 2022). For both groups in the present study, the rates of success were higher than those previously reported in the literature (DCM (42-71%) (Mjåset et al., 2022; Evaniew et al., 2023; Dijkman et al., 2022; Tetreault et al., 2019) and radiculopathy patients (69%) (Mjåset et al., 2020)), although the precise values reported may depend on how success was defined in each case.

The proportion of good-GTO stayed relatively constant over the whole FU duration, indicating that, for all degenerative diagnoses included, the “success rate” of surgery as self-assessed by the patients remained stable between 3mo and 5y, as has also been observed in a study on various degenerative disorders of the cervical spine (Buttermann, 2018) or in another DCM cohort up to their final 2-year FU (Evaniew et al., 2023).

### 4.5 Implications from all 3 outcomes

This study assessed the outcomes of DCM patients and a matched control group using 3 different PROMs, whose patterns of change were not always entirely consistent with each other. For instance, there were no significant group differences for COMI and PASS but there were for GTO. Also, while the proportions of patients reporting a good GTO were relatively stable over the whole FU duration, the COMI score and proportion of patients in PASS varied slightly over time of FU. There are various potential explanations for this.

First, while the 3 different scales are all outcome measures self-rated by the patients, they are intrinsically different and do not appraise identical constructs: the COMI assesses the current burden of the disease across multiple domains as a *continuous score*; the PASS enquires if a patient feels *well/bad* in their current state; the GTO in turn asks if a patient is feeling *better/worse* compared to the pre-operative state. A second possible explanation could be related to the response shift phenomenon (Tetreault et al., 2019; Schwartz et al., 2009)). DCM patients, because of the spinal cord damage inherent to their condition, suffer more from functional impairments than patients with other degenerative disorders of the cervical spine, who encounter more pain (McCartney et al., 2018). Previous studies have observed that patients tend to get used to functional impairments – but not to pain – and postulated that patients suffering from functional decline gradually recalibrate their internal standards and expectations as they live with their progressing condition. Because of this recalibration, they tend to underestimate their pre-operative level of impairment (Schwartz et al., 2009; Tubach et al., 2006). This might in turn explain why the extent of improvement between the current post-operative state and the pre-operative state (measured by the GTO) would not be rated as highly by the DCM patients as by the control patients —despite similar prospectively measured COMI scores and PASS proportions — and thus decrease the number of patients with a good self-rated GTO. Third, expectations and recall bias have also been reported to differ between DCM patients and patients suffering from other degenerative disorders of the cervical spine (Krauss et al., 2022), (Mancuso et al., 2016), which might also explain differences in patient-rated outcomes between the groups. Finally, some would argue that the COMI, not having been specifically designed for DCM patients, might not be sensitive enough to assess some of the impairments caused by DCM and might not appraise accurately the burden of the disease. It is worth noting that none of the three measures used in this study were specifically developed for DCM. However, the GTO did identify relevant differences, whereas the PASS, which is also not specific to a given symptom, would have been expected to be sufficiently sensitive (Mannion et al., 2015, 2016) but showed no significant group differences.

None of the aforementioned factors could be directly evaluated with our data and they should be the goal of future studies that are specifically designed to better understand their role, mechanisms, and relative weight.

### 4.6 Strengths

A strength of the current study is that the DCM and control cohort were propensity-score matched using a constellation of baseline variables, thus limiting selection bias and allowing more accurate comparisons across the groups. Another advantage is the large number of patients that even after matching resulted in a dataset of 708 cases with no strict exclusion criteria or specific surgical technique, thus allowing the generalization of results and their application to other centres and regions (Dijkman et al., 2022). The large sample size excludes the possibility that type II errors were responsible for the lack of group differences observed.

### 4.7 Limitations

Some limitations should be mentioned. First, this study was a retrospective analysis of prospectively collected data and therefore has a relatively low level of evidence. Second, only patients having filled out the questionnaire at all 5 time points were included in the analysis and, while the compliance at all time points was high in both groups (>68% at 5 years, and >81% at all other time points), only 68.5% of control and 58.4% of DCM patients had a complete set of forms. Although these compliance rates are considered acceptable for an institutional registry (van Hooff, 2015) and the set of patients analysed was very large, attrition bias (and a differing level of attrition bias between the groups) cannot be ruled out. The lower compliance in DCM patients was due to administrative reasons not attributable to their specific diagnosis, as well as a higher mortality before the end of FU, although the cohorts were matched for age, comorbidity and invasiveness of surgery. The higher mortality may be the result of some patients in the DCM group having had more severe DCM disease at baseline, rendering them more likely to have a shorter life expectancy than the general population or patients with mild DCM symptoms, even after surgery (Davies et al., 2023). Third, although, propensity-score matching balanced most baseline differences between the groups, a small but statistically significant imbalance remained in surgical approach, with the DCM cohort having a higher proportion of posterior and combined procedures. Since the latter two approaches are usually favoured in more complex cases and surgeries involving multiple spinal levels (Kim et al., 2025), the imbalance could be a sign of confounding by indication. However, the poorer post-operative recovery that has been linked to these approaches is time-limited and tends to diminish over longer-term follow-up, with outcomes converging by approximately 2 years (Kouli et al., 2026). In the present study, COMI and PASS demonstrated similar trajectories in both groups (*Group* factor not significant), while GTO remained consistently lower in the DCM cohort at all time points (*Group* significant but not the *Group∗Time* interaction). Therefore, although the imbalance in surgical approach may have influenced early post-operative outcomes, it is unlikely to fully account for the persistent differences in GTO observed over time.

Finally, the control group was analysed as a whole and not in relation to their main pathologies. It cannot be ruled out that some subcategories of the patient cohort may have had different outcomes over time. This would need to be the scope of another study.

## 5 Conclusions

This study showed that patients with DCM and a matched cohort of patients with other types of cervical spine degenerative disease improved considerably after surgery. The evolution over time of all patient-rated outcomes was similar in both groups, meaning that the average rate of recovery was comparable. However, while the prospectively recorded outcomes did not differ between the groups, patients with DCM gave significantly worse ratings for the global treatment outcome (how much the operation had helped their problem) than did the control group. Further studies should investigate the reasons underlying this discrepancy.

## Authorship confirmation statement

All authors contributed to the study conception. The data collection and analysis were performed by FM, FG, AC and AFM. The manuscript was drafted by FM and revised by AFM, FG, AC and TFF. All authors read and approved the final manuscript.

## Ethical consideration

Ethics approval (BASEC-Nr. 2023-01683) was obtained for the re-use of routinely collected data for research purposes, from patients who gave their signed informed consent for this.

## Authors’ disclosure statement

The authors declare no potential conflicts of interest with respect to the research, authorship, and/or publication of this article.

## Data availability

Data used to support the findings of this study are available from the corresponding author upon reasonable request.

## Funding statement

This study received no funding.

## Declaration of competing interest

The authors declare that they have no known competing financial interests or personal relationships that could have appeared to influence the work reported in this paper.
